# Narrowband Internet of Things via Low Earth Orbit Satellite Networks: An Efficient Coverage Enhancement Mechanism Based on Stochastic Geometry Approach

**DOI:** 10.3390/s24062004

**Published:** 2024-03-21

**Authors:** Tao Hong, Xiao Yu, Ziwei Liu, Xiaojin Ding, Gengxin Zhang

**Affiliations:** School of Communications and Information Engineering, Nanjing University of Posts and Telecommunications, Nanjing 210003, China; 1221013826@njupt.edu.cn (X.Y.); lzw@njupt.edu.cn (Z.L.); dxj@njupt.edu.cn (X.D.); zgx@njupt.edu.cn (G.Z.)

**Keywords:** Internet of Things (IoT), NB-IoT, LEO satellite IoT networks, coverage enhancement, random access, stochastic geometry theory

## Abstract

With the development of IoT technology and 5G massive machine-type communication, the 3GPP standardization body considered as viable the integration of Narrowband Internet of Things (NB-IoT) in low Earth orbit (LEO) satellite-based architectures. However, the presence of the LEO satellite channel comes up with new challenges for the NB-IoT random access procedures and coverage enhancement mechanism. In this paper, an Adaptive Coverage Enhancement (ACE) method is proposed to meet the requirement of random access parameter configurations for diverse applications. Based on stochastic geometry theory, an expression of random access channel (RACH) success probability is derived for LEO satellite-based NB-IoT networks. On the basis of a power consumption model of the NB-IoT terminal, a multi-objective optimization problem is formulated to trade-off RACH success probability and power consumption. To solve this multi-objective optimization problem, we employ the Non-dominated Sorting Genetic Algorithms-II (NSGA-II) method to obtain the Pareto-front solution set. According to different application requirements, we also design a random access parameter configuration method to minimize the power consumption under the constraints of RACH success probability requirements. Simulation results show that the maximum number of repetitions and back-off window size have a great influence on the system performance and their value ranges should be set within [4, 18] and [0, 2048]. The power consumption of coverage enhancement with ACE is about 58% lower than that of the 3GPP proposed model. All this research together provides good reference for the scale deployment of NB-IoT in LEO satellite networks.

## 1. Introduction

With the widespread adoption and advancement of low-power wide-area network technology, the deployment and connectivity demands for Internet of Things (IoT) devices have experienced an exponential surge. Challenges arise in regions such as oceans, deserts, and remote mountainous areas, where constructing terrestrial-based stations is impractical or cost-prohibitive. As a consequence, Narrowband Internet of Things (NB-IoT) terminals relying solely on terrestrial-based infrastructure exhibit notable deficiencies in service capacity, leading to a significant disparity between service capabilities and actual demands [1]. In response to these challenges, low Earth orbit (LEO) satellite-based IoT networks have emerged as a promising solution, serving as a complementary and extended alternative to terrestrial-based networks. LEO satellite-based IoT networks address the limitations associated with deploying terrestrial-based stations and establishing communication networks on the ground [2]. Iridium Communications announced Project Stardust which it describes as “the evolution of its direct-to-device (D2D) strategy with 3GPP 5G standards-based NB-IoT via non-terrestrial network (NTN) systems [3]. This approach boasts advantages such as global coverage and the ability to deploy terminals without substantial spatial constraints. Therefore, LEO satellite-based IoT networks have become a hot topic in the IoT research field.

The application of NB-IoT as a public network IoT system in LEO satellite-based networks has been widely studied, with a primary focus on the adaptive modifications of NB-IoT in the context of high-dynamic and long-distance transmission links in LEO satellite channels, such as waveform, synchronization, protocol stack, etc. In [4], the authors focus on the impact of Doppler shift on NB-IoT waveforms and propose some possible solutions. In the literature [5], the authors not only take into account the adaptations at the NB-IoT physical layer but also include the higher layers, like Medium Access Control (MAC), Radio Link Control (RLC), Packet Data Convergence Protocol (PDCP), and Radio Resource Control (RRC) layers, in LEO satellite-based networks. The authors propose a resource allocation approach in order to reduce the high values of differential Doppler under the maximum value supported by the 3GPP standard. They also demonstrate the link budgets of LEO satellite-based networks with different parameters, providing some simulation results as a benchmark for further study. To address the problem during the RA procedure, they investigate the effects of the differential Doppler and delay on the RA procedure and point out some solutions to overcome those challenges, such as the use of Global Navigation Satellite System (GNSS)-aided solutions and the removal of Hybrid Automatic Repeat Request (HARQ). In addition, they propose an RA technique which is robust to typical satellite channel impairments. Simulation results show that the proposed method can address the problem of long delay in an NTN scenario. In [6], the authors review the fundamentals of NB-IoT and explain how to support NB-IoT satellite communication through a minimal set of modifications. In [7], the authors select the suitable antenna and proper satellite parameters to ensure link reliability in LEO satellite-based IoT networks. In [8], the authors describe the NB-IoT uplink problem in LEO satellite-based networks and increase the compensation range of the NB-IoT receiver by adding the number of Demodulation Reference Signal (DMRS) symbols. Furthermore, the RA procedure is the main challenge of NB-IoT technology’s application into the NTN scenario due to the presence of a long delay. Therefore, more researchers have focused on the problems during the RA procedure in LEO satellite-based IoT networks.

To solve the problems, the authors in [9] analyze the challenges imposed by the increased delay in the communication link on the RA procedure and propose new solutions to overcome those challenges. To extend the supported satellite beam size, the authors in [10] design a new preamble format for the NB-IoT RA procedure. The work in [11] presents a simulation tool which is conceived as a new module for the open-source 5G-air-simulator, deriving the performance of NB-IoT in LEO satellite-based networks. The authors in [12] make a comparison of the RACH success probability and the access time for different combinations of the configurable random access parameters which are given by the 3GPP standard [13]. Although the studies mentioned above have proposed some solutions to the problems that may arise in the NB-IoT RA procedure, the impact of the NB-IoT unique coverage enhancement (CE) mechanism in LEO satellite-based networks has not been taken into consideration.

In the NB-IoT RA procedure, 3GPP mandated a special link adaptation technology named the CE mechanism to achieve the objective of the 20 dB enhanced coverage of NB-IoT for wide-area outdoor coverage [14]. However, the NB-IoT terminal distributed in the center and edge of the LEO satellite beam has a large difference in the Signal-to-Interference-plus-Noise Ratio (SINR) due to the wide coverage of LEO satellite beams. Therefore, it is crucial to ensure lower power consumption and diverse business RACH success probability requirements in an NTN scenario [15]. The CE mechanism plays a key role in meeting these demands. If we directly employ the CE parameter configuration of terrestrial-based networks in LEO satellite-based IoT networks, it will result in lower RACH success probability and higher power consumption because of the long propagation delay between LEO satellites and NB-IoT terminals. 

By addressing the above challenges, the major contributions in this paper are summarized as follows:

- To address the problem of CE parameter configurations in LEO satellite-based IoT networks, we propose an Adaptive Coverage Enhancement (ACE) method to optimize RACH success probability and the power consumption of the terminal for diverse LEO satellite-based IoT applications.
- Based on stochastic geometry theory, we present a mathematical framework for analyzing RACH success probability in the LEO satellite-based IoT networks and derive a closed-form expression of RACH success probability for NB-IoT terminals.
- To meet the different requirements of diverse applications in LEO satellite-based IoT networks, we formulate an optimization problem, that considers the trade-off between RACH access success probability and power consumption, to optimize the number of maximum repetitions and back-off window size. This is essentially a multi-objective optimization problem. To solve this optimization problem, we employ the Non-dominated Sorting Genetic Algorithms-II (NSGA-II) method to obtain the Pareto front for different applications. On this basis, we also present a parameter configuration method to achieve the RACH parameter settings of the NB-IoT terminal.
- In order to evaluate the performance of proposed ACE method by system-level simulation, we use the NS-3 simulator to simulate the system throughput and support NB-IoT’s technical application in LEO satellite-based IoT networks.

The rest of this paper is organized as follows. Section 2 describes the system model and the principle of CE. In Section 3, we derive the RACH success probability and establish a power consumption model for NB-IoT in a LEO satellite channel. In Section 4, we formulate the optimization problem and solve it to obtain RA parameters for diverse applications. Simulation results are presented in Section 5. Finally, we conclude this paper in Section 6.

## 2. System Model

In this section, we present a system architecture and channel model of LEO satellite-based NB-IoT networks. We also illustrate the principles of the RA procedure in an NTN scenario. On this basis, we focus on analyzing the problem of the CE mechanism in LEO satellite-based IoT networks.

### 2.1. NB-IoT via LEO Satellite-Based Networks

**Figure 1:**
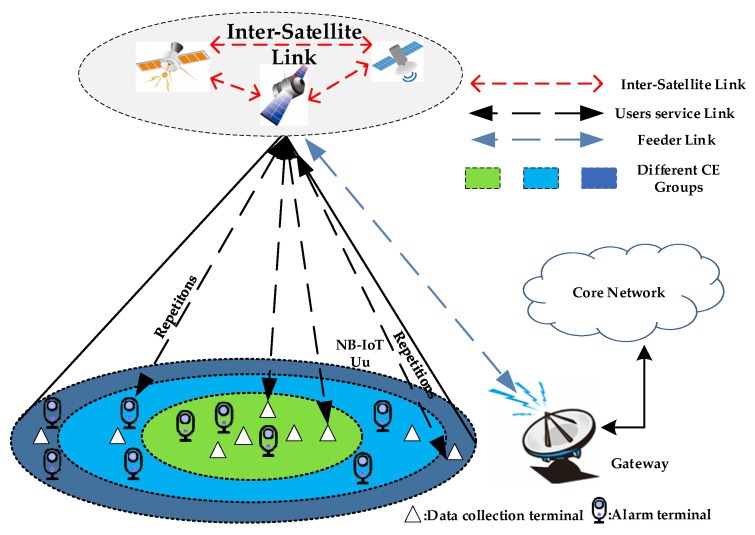
System architecture of LEO satellite-based NB-IoT networks.

Figure 1 shows a system architecture of LEO satellite-based IoT networks in remote areas, which consists of multiple NB-IoT terminals, LEO satellites, and a gateway station. The LEO satellite transponder is equipped with a regenerative transponder. In this way, the onboard demodulation of data packets is executed by the LEO satellite itself. When the gateway station is not visible to the current service satellite, data packets will then be transferred to other LEO satellites in the gateway station’s line of sight through Inter-Satellite Links (ISLs). Subsequently, the data packets are further transmitted to the gateway station through the feeder link within the line of sight of the gateway station. We consider that each NB-IoT terminal is equipped with a GNSS receiver to pre-compensate Doppler shift and time advance (TA), which are caused by the high dynamics and long delay of the LEO satellite channel. This is possible thanks to the knowledge of satellite ephemeris and available terminal locations. Depending on the prediction accuracy, additional delay and frequency offsets are assumed to be handled by the pre-compensation. In addition, NB-IoT terminals are divided into different CE groups according to their downlink Reference Singal Receiving Power (RSRP) measurement. For the different RACH success probability requirement applications, such as data collection, asset tracking, and alarm traffic, the NB-IoT terminal needs suitable RACH parameter settings to optimize the power consumption. In this paper, we focus on the relationship between RACH success probability and the power consumption of the NB-IoT terminal for different IoT applications.

### 2.2. LEO Satellite Channel Model

Without a loss of generality, the LEO satellite channel model takes into account the large-scale fading with path loss exponent α. Further, the small-scale fading follows the Rician fading. The probability distribution function (PDF) of channel power gain can be given by the following:

(1) f|h0|2=ae−βxF11(ms;1;δx)

where $a=\frac{1}{2b}{\left(\frac{2bm_{s}}{2bm_{s}+\Omega_{s}}\right)}^{m_{s}}$, $\beta =\frac{1}{2b}$, $\delta =\frac{\Omega_{s}}{2b\left(2bm_{s}+\Omega_{s}\right)}$, $\Omega_{s}$ is the average power of the line-of-sight path, 2b is the average power of the scattered path, $m_{s}$ is the fading severity parameter, and F11 is the confluent hypergeometric function.

It is noteworthy that the interfering terminals located at the beam edge have smaller elevation angles caused by the wide coverage of the LEO satellite beam. The smaller elevation angles result in a low K factor, which will change the Rician distribution into a Rayleigh distribution. Therefore, we consider that the Rayleigh fading model is applicable for the interfering terminal with a low-probability line-of-sight (LOS) channel at the edge of the LEO satellite beam.

We have compared our channel model with the series of models presented in the article [16]. In the article, they have collected 13 different models for NGSO satellites where each one of them highlights a predominant channel feature. Among them, Model 1 conforms to our channel modeling. Model 1 divides the channel model into two states: the good state means that terminals correspond to unshaded areas with high received signal power or the LOS component which is represented by Rician statistical distributions fading; the Bad state means that terminals correspond to areas with low received signal power also called non-LOS (NLOS) which is represented by Rayleigh or log-normal distribution fading. In our paper, we also divide our channel model into two situations: For NB-IoT terminals located at the center of the satellite beam, they can have stronger LOS components. Due to the presence of the direct path, the small-scale fading of the channel model for these terminals is more in line with the Rice distribution. NB-IoT terminals located at the beam edge have smaller elevation angles caused by the wide coverage of the LEO satellite beam. The Rayleigh fading model is applicable when the LOS portion of signals received at the terminal’s place is smaller than that of NLOS components.

### 2.3. The Random Access Procedure and Coverage Enhancement

**Figure 2:**
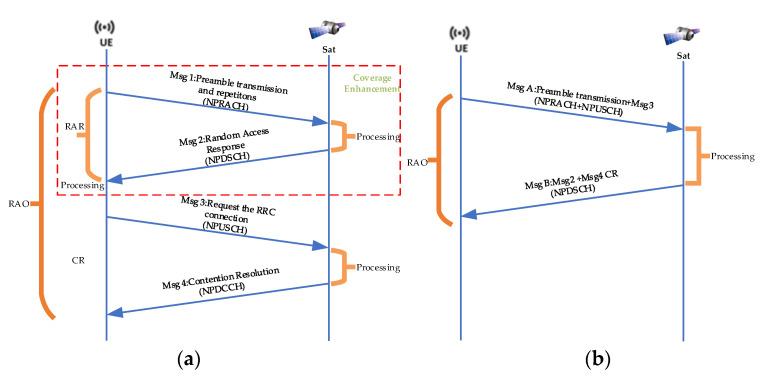
Random access procedure: (**a**) four-step random access procedure; (**b**) two-step random access procedure.

Figure 2 shows the RA procedure between the terminal and LEO satellite. For LEO satellite-based IoT networks, a two-step random access procedure can reduce half of the access delay at the expense of receiver complexity compared with four-step random access [17]. Without a loss of generality, we analyzed the RACH success probability on the basis of the four-step random access procedure, because four-step random access is the 3GPP recommended standard [18] in NTN networks. Furthermore, we focus on repetitions of the preamble for achieving the CE mechanism in MSG1 and the random access response (RAR) in MSG2 on the condition that MSG3 and MSG4 are in ideal states and always successful. It is important to note that the most critical part of the RA procedure is the repetition of the preamble in the MSG1 because the terminal has not synchronized with the networks [19]. Therefore, we assume that if the MSG1 and MSG2 processes are successful in transmission, then the entire random access procedure is considered successful in this paper.

The two key factors affecting the RACH success probability are as follows: (1) whether the SINR of the preamble received by the LEO satellite is above the demodulation threshold; (2) whether multiple NB-IoT terminals select the same preamble in the same time slot. Combining the probabilities of these two key factors, we derive the RACH success probability in Section 3.

**Figure 3:**
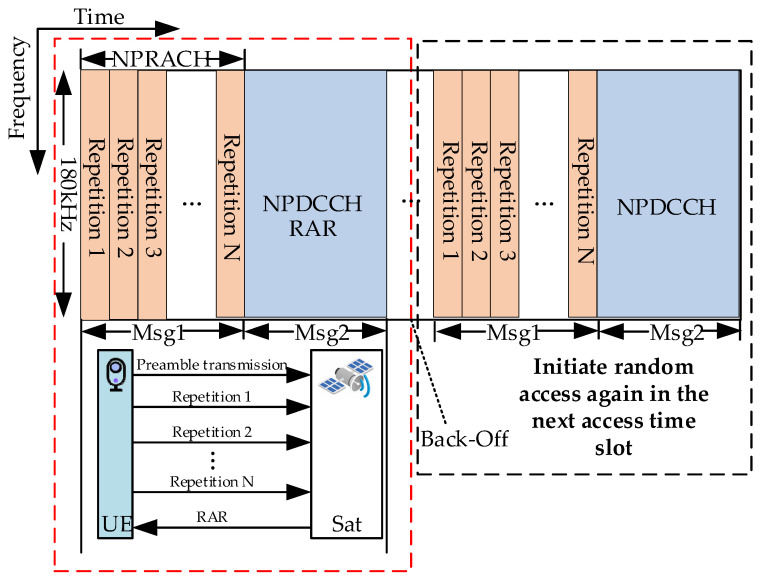
Example of NB-IoT coverage enhancement mechanism.

Figure 3 illustrates the schematic diagram of the NB-IoT CE mechanism in LEO satellite-based IoT networks. From the figure, it can be seen that the CE mechanism is mainly realized by the repetition of the preamble. NB-IoT terminals measure the value of RSRP to determine its initial coverage class. In this way, NB-IoT terminals select appropriate random access parameters to match the coverage class. When a terminal fails to receive the RAR sent by the LEO satellite, it enters a back-off state and restarts the RA procedure again in the next time slot. Although repetitions increase the transmission delay, it can enhance the processing gain of the receiver and extend the coverage of the LEO satellite. Consequently, the CE mechanism is a crucial function for NB-IoT to achieve wide-area coverage in LEO satellite-based IoT networks.

Table 1 shows the NB-IoT repetition parameter configuration in the terrestrial networks. Because the distance between ground base stations and NB-IoT terminals is generally within a few thousand meters, the delay caused by repetitions to the terrestrial networks can be ignored. Conversely, the transmission delay must be considered in the RA procedure due to the fact that the value ranges from 2 ms to 5 ms during the overhead period of a LEO satellite with 600 km height. Moreover, the transmission delay exhibits significant differences when the NB-IoT terminal is distributed at the center and edge of the LEO satellite beam. In summary, if we use the random access parameters of the terrestrial networks, the repetition of the preamble will cause the NB-IoT terminal to be always in the state of transmitting MSG1 due to the existence of a long delay and the high dynamics of the LEO satellite. This situation not only fails to establish a connection with the LEO satellite but also results in a lot of power consumption. Furthermore, the choice of an excessively long back-off window size is similarly unfavorable to the RA procedure in LEO satellite-based IoT networks. A long back-off window size means that the terminal has to be in the RRC Connected state for a long time which will increase power consumption. It can also lead to a channel state of outdated information when the RA procedure restarts in the next time slot. The outdated CSI will cause a loss of synchronization and result in access failure. Therefore, we need optimize the number of repetitions and the size of the back-off window to meet the requirements of NB-IoT terminals in the LEO satellite IoT networks. 

## 3. RACH Success Probability and Power Consumption Model for LEO Satellite-Based NB-IoT Networks

In this section, we derive the RACH success probability based on stochastic geometry theory in LEO satellite-based networks. Furthermore, we also analyze the power consumption of NB-IoT terminals using repetition to achieve the CE mechanism.

### 3.1. Interference and Collision Model

Without a loss of generality, we consider that the number of the data packet received by the LEO satellite in the coverage area is a random variable following the Poisson distribution and the arrival rate is λ. Therefore, the probability density function (PDF) is written as follows:

(2) $$P\left(X=k\right)=\frac{e^{-\lambda }}{k!}\lambda^{k}$$

In order to provide the expressions for the preamble detection success probability in the following parts, we assume that NB-IoT terminals’ distribution follows a homogeneous Poisson point process (HPPP) to analyze the SINR. The locations of them are distributed according to a homogeneous HPPP [20]. The PDF of the distance from the LEO satellite to the NB-IoT terminal can be given by the following:

(3) $$f_{R}\left(r\right)=\left\{ \begin{array}{l} \frac{r}{2R_{e}\left(R_{e}+r_{\min}\right)}\;r_{\min}\leq r\leq 2R_{e}+r_{\min}\;\\ 0\;\;\;\;\;otherwise \end{array}\right.\;$$

where $R_{e}$ is Earth’s radius, $r_{\min}$ specifies the minimum possible distance from a satellite to a terminal (that is realized when it is directly above the terminal), and $r_{\max}=\sqrt{2R_{e}\cdot r_{\min}+r_{\min}^{2}}$ is the maximum possible distance (that is realized when the LEO satellite is at the horizon).

**Figure 4:**
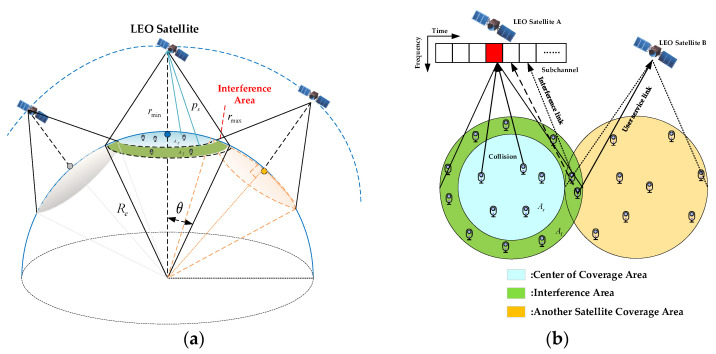
Interference model: (**a**) interference model architecture diagram; (**b**) top view of interference area.

Figure 4a shows the interference model of NB-IoT via LEO satellite-based networks. NB-IoT terminals initiate the RA procedure with the same transmit power $p_{s}$. We consider that the LEO additive noise of the LEO satellite is subject to additive white Gaussian noise (AWGN) with constant power $\sigma^{2}$. Moreover, the LEO satellite antenna receiving gain $G_{0}$ satisfies the following equation [21]:

(4) $$G_{0}=\eta {\left(\frac{\pi D}{\lambda }\right)}^{2}=\eta {\left(\frac{f\pi D}{c}\right)}^{2}$$

where η is aperture efficiency, D is reflective surface aperture, f is the frequency band, and c is the speed of light.

As shown in Figure 4b, when NB-IoT terminals are in the overlapping region of the beam coverage of LEO satellite A and LEO satellite B, the downlink RSRP received by NB-IoT terminals in LEO satellite B will be similar to the RSRP broadcast from LEO satellite A. Therefore, this situation will result in NB-IoT terminals which should connect to LEO satellite B causing interference to LEO satellite A. Due to the presence of satellite constellation, the interference area $A_{I}$ can be approximated as a circle. Meanwhile, collisions may occur when NB-IoT terminals are in the same LEO satellite coverage area $A_{s}$. Given the above, we will analyze the successful probability of preamble detection and the probability of preamble collision in the following subsection.

### 3.2. The Successful Probability of Preamble Detection

Based on the interference model, the SINR of the LEO satellite receiving a preamble signal can be expressed as the following:

(5) $$SINR=\frac{p_{s}{\left|h_{0}\right|}^{2}G_{0}R_{0}^{-\alpha }}{I_{m}+\sigma^{2}}$$

where $I_{m}$ is the cumulative interference from all other terminals in the interference area $A_{I}$ and can be given by:

(6) $$I_{m}=\sum_{m=1}^{M_{I}}p_{s}{\left|h_{m}\right|}^{2}G_{0}R_{m}^{-\alpha }$$

where $M_{I}$ is a random variable representing the terminal causing interference, ${\left|h_{m}\right|}^{2}$ is the channel power gain of Rayleigh fading, and $p_{s}$ is the transmission power of the interfering terminal.

If the SINR is higher than the LEO satellite demodulation threshold $\gamma_{th}$, the LEO satellite can successfully detect the preamble. The probability of successful preamble detection can be expressed as follows:

(7) $$\begin{array}{l} p_{c}=\mathbb{P}\left(SINR\geq \gamma_{th}\right)\\ \;=E_{r}[f\{SINR\geq \gamma_{th}|r_{0}\}]\\ \;=\int_{r\min}^{r\max}f_{R}(r)f(SINR\geq \gamma_{th}|r_{0})dr_{0}\\ \;=\int_{r\min}^{r\max}f_{R}(r)f(\frac{p_{s}{\left|h_{0}\right|}^{2}G_{0}{r_{o}}^{-\alpha }}{\sigma^{2}+I_{m}}\geq \gamma_{th}|r_{0})dr_{0}\\ \;=\int_{r\min}^{r\max}\frac{r_{0}}{2R_{e}(R_{e}+r_{\min})}f[G_{0}\geq \gamma_{th}{r_{0}}^{\alpha }{\left|h_{0}\right|}^{2}{p_{s}}^{-1}\left(\sigma^{2}+I_{m}\right)|r_{0}]dr_{0} \end{array}$$

By applying the Laplace transform to the set of interfering terminals, the expression for the successful probability of preamble detection is given by the following:

(8) $$\begin{array}{l} f[G_{0}\geq \gamma_{th}{r_{0}}^{\alpha }{\left|h_{0}\right|}^{2}{p_{s}}^{-1}\left(\sigma^{2}+I_{m}\right)|r_{0}]\\ \;\;\;\;\;=E_{I_{m}}\left[f[G_{0}\geq \gamma_{th}{r_{0}}^{\alpha }{\left|h_{0}\right|}^{2}{p_{s}}^{-1}\left(\sigma^{2}+I_{m}\right)|r_{0},I_{m}]\right]\\ \;\;\;\;\;=E_{I_{m}}\left[\exp\left(-\gamma_{th}r_{0}^{\alpha }{\left|h_{0}\right|}^{2}{p_{s}}^{-1}\left(\sigma^{2}+I_{m}\right)\right)|r_{0}\right]\\ \;\;\;\;\;=e^{-\gamma_{th}{r_{0}}^{\alpha }{\left|h_{0}\right|}^{2}p_{s}^{-1}\sigma^{2}}L_{I_{m}}(\gamma_{th}r_{0}^{\alpha }{p_{s}}^{-1}) \end{array}$$

Substituting Equation (8) into Equation (7), we obtain the following expression:

(9) $$p_{c}=\int_{r\min}^{r\max}\frac{r_{0}}{2R_{e}(R_{e}+r_{\min})}e^{-\gamma_{th}{r_{0}}^{\alpha }{\left|h_{0}\right|}^{2}p_{s}^{-1}\sigma^{2}}L_{I_{m}}(\gamma_{th}r_{0}^{\alpha }{p_{s}}^{-1})dr_{0}$$

where $L_{I_{m}}(\gamma_{th}r_{0}^{\alpha }{p_{s}}^{-1})$ represents the Laplace transform of the interference terminals. And let $s=\gamma_{th}r_{0}^{\alpha }{p_{s}}^{-1}$ to solve the Laplace transform of the interference terminals.

(10) $$\begin{array}{l} L_{I_{m}}\left(s\right)=E_{I_{m}}\left(e^{-sI_{m}}\right)\\ \;\;\;=E_{I_{m}}\left(\exp\left(-\sum_{M=1}^{M_{I}}sp_{s}G_{0}R_{m}^{-\alpha }\right)\right)\\ \;\;\;=E_{G_{0}D_{m}}\left[\prod_{M\in M_{I}}\exp(-sp_{s}G_{0}R_{m}^{-\alpha })\right] \end{array}$$

Since $G_{0}$ is a constant variable, we can derive the following:

(11) $$L_{I_{m}}\left(s\right)=E_{D_{m}}\left[\prod_{n\in N_{I}}E_{G_{0}}\exp(-sp_{s}G_{0}R_{m}^{-\alpha })\right]$$

Let $R_{m}=x$ represent the distance between the interference terminals and the LEO satellite. By utilizing the PDF from stochastic geometry theory [22], we can obtain the following result:

(12) $$\begin{array}{l} L_{I_{m}}\left(\gamma_{th}r_{0}^{\alpha }{p_{s}}^{-1}\right)=\exp\left[-2\pi \lambda \int_{r0}^{r_{\max}}\frac{1}{1+{\left(\gamma_{th}r_{0}^{\alpha }\right)}^{-1}x^{\alpha }}xdx\right]\\ \;\;\;\;\;\;=\exp\left[-2\pi \lambda \int_{r0}^{r_{\max}}\frac{\gamma_{th}}{\gamma_{th}+{\left(x/r_{0}\right)}^{\alpha }}xdx\right] \end{array}$$

By substituting Equation (12) into Equation (9), we can obtain the expression for the probability of successful preamble detection:

(13) $$p_{c}=\int_{r\min}^{r\max}\frac{r_{0}\cdot e^{-\gamma_{th}{r_{0}}^{\alpha }{\left|h_{0}\right|}^{2}p_{s}^{-1}\sigma^{2}}}{2R_{e}(R_{e}+r_{\min})}\exp\left(-2\pi \lambda \int_{r0}^{r_{\max}}\frac{\gamma_{th}}{\gamma_{th}+{\left(x/r_{0}\right)}^{\alpha }}xdx\right)dr_{0}$$

As has been noted, we have derived the probability of successful preamble detection for NB-IoT via LEO satellite-based networks based on stochastic geometry theory. Moreover, one of the techniques used in the NB-IoT CE mechanism is the repetition of the preamble. The probability of successful preamble detection $p_{cd}$ can be expressed as follows:

(14) $$\begin{array}{l} p_{cd}=1-\left(1-p_{c}\right)p_{\varepsilon }\\ \;\;=1-\left(\int_{r\min}^{r\max}\frac{r_{0}\cdot e^{-\gamma_{th}{r_{0}}^{\alpha }{\left|h_{0}\right|}^{2}p_{s}^{-1}\sigma^{2}}}{2R_{e}(R_{e}+r_{\min})}\exp\left(-2\pi \lambda \int_{r0}^{r_{\max}}\frac{\gamma_{th}}{\gamma_{th}+{\left(x/r_{0}\right)}^{\alpha }}xdx\right)dr_{0}\right)e^{-\left(N_{rep}-1\right)} \end{array}$$

where $p_{\varepsilon }$ represents the reduction factor which is influenced by the LEO satellite capture effect [23] and $N_{rep}$ denotes the number of repetitions of the preamble.

### 3.3. RACH Success Probability Analysis

As mentioned above, the traffic model of NB-IoT terminals is modeled as a Poisson distribution. On this basis, we give the probability of preamble collision and derive the RACH success probability in LEO satellite-based networks. 

We consider that $N_{acc}$ NB-IoT terminals participate in the RA procedure to contend S available preambles. Therefore, the number of NB-IoT terminals $N_{s}$ that successfully access the network in this RA procedure can be given by the following:

(15) $$N_{s}=N_{acc}\cdot e^{\frac{N_{acc}}{S}}$$

In the ith RA procedure, the number of NB-IoT terminals which have the nth repetition can be denoted by the following:

(16) $$N_{i}=\sum_{n=1}^{N_{rep}^{\max}}N_{i}\left[n\right]$$

where $N_{rep}^{\max}$ is the maximum number of repetitions. 

The number of NB-IoT terminals that successfully and unsuccessfully acquire access in the ith RA procedure can be derived by Equations (15) and (16), respectively, as shown in Equations (17) and (18):

(17) $$N_{i}^{s}=N_{i}\cdot e^{-\frac{N_{i}}{S}}=\sum_{n=1}^{N_{rep}^{\max}}\left(N_{i}\left[n\right]\cdot e^{-\frac{N_{i}}{S}}\right)$$

(18) $$N_{i}^{f}=N_{i}\cdot \left(1-e^{-\frac{N_{i}}{S}}\right)=\sum_{n=1}^{N_{rep}^{\max}}\left(N_{i}\left[n\right]\cdot \left(1-e^{-\frac{N_{i}}{S}}\right)\right)$$

If data packet collision occurs, the NB-IoT terminal will restart the RA procedure in the next time slot after the back-off window. Due to the different sizes of the back-off windows, the terminal starts the RA procedure in a different time slot. Without a loss of generality, we consider that the uniform back-off strategy is employed, and the probability of different terminals’ back-off to a time slot is the same. The probability can be denoted as P:

(19) $$P=\frac{1}{Len}=\left[\frac{T_{\mathrm{RAO}}}{W_{BO}+T_{RAR}+RTD+\mathrm{StartRAR}}\right]$$

where Len is the maximum number of RA procedures that NB-IoT terminals can restart during a RAO, $W_{BO}$ is the size of the back-off window, $T_{RAR}$ is the size of the RAR window, RTD signifies the Round-Trip Delay (RTD), and StartRAR denotes the active timer of the waiting RAR which value is 41 ms in the NTN scenario.

When n > 1, $N_{i}\left[n\right]$ represents the number of NB-IoT terminals that start the ith RA procedure which can be expressed as follows:

(20) $$N_{i}[n]=P\cdot \sum_{k}^{Len}N_{i-k}^{f}\left[n-1\right]\;\;2\leq n\leq N_{\mathrm{rep}}^{\max}$$

From this equation, we can deduce the number of NB-IoT terminals $N_{i}^{s}\left[n\right]$ which successfully acquire access in the nth random access procedure as follows:

(21) $$N_{i}^{s}[n]=N_{i}\left[n\right]\cdot e^{-\frac{N_{i}}{S}}$$

Therefore, the probability of successful access can be given by the following:

(22) $$\begin{array}{l} p_{u}=\frac{\sum_{i=1}^{T_{\max}}N_{i}^{s}}{N_{acc}}=\frac{\sum_{i=1}^{T_{\max}}\sum_{n=1}^{N_{\mathrm{rep}}^{\max}}N_{i}^{s}\left[n\right]}{N_{acc}}=\frac{\sum_{i=1}^{T_{\max}}\sum_{n=1}^{N_{\mathrm{rep}}^{\max}}\sum_{k=1}^{Len}P\cdot N_{i-k}^{f}[n-1]\cdot e^{-N_{i}/S}}{N_{acc}}\\ \;=\frac{\sum_{i=1}^{T_{\max}}\sum_{n=1}^{N_{\mathrm{rep}}^{\max}}\sum_{k=1}^{Len}\frac{W_{BO}+T_{RAR}+\mathrm{RTD}+41}{T_{RAO}}\cdot N_{i-k}^{f}[n-1]\cdot e^{-N_{i}/S}}{N_{acc}} \end{array}$$

According to aforementioned analysis, the RACH success probability can be ex-pressed by the following:

(23) $$\begin{array}{l} p_{s}=p_{cd}\cdot p_{u}\\ \;=\left(1-\left(\int_{r\min}^{r\max}\frac{r_{0}\cdot e^{-\gamma_{th}{r_{0}}^{\alpha }{\left|h_{0}\right|}^{2}p_{s}^{-1}\sigma^{2}}}{2R_{e}(R_{e}+r_{\min})}\exp\left(-2\pi \lambda \int_{r0}^{r_{\max}}\frac{\gamma_{th}}{\gamma_{th}+{\left(x/r_{0}\right)}^{\alpha }}xdx\right)dr_{0}\right)e^{-\left(N_{rep}-1\right)}\right)\\ \;*\frac{\sum_{i=1}^{T_{\max}}\sum_{n=1}^{N_{\mathrm{rep}}^{\max}}\sum_{k=1}^{Len}\frac{W_{BO}+T_{RAR}+\mathrm{RTD}+41}{T_{RAO}}\cdot N_{i-k}^{f}[n-1]\cdot e^{-N_{i}/S}}{N_{acc}} \end{array}$$

### 3.4. Power Consumption Model

In this subsection, we establish a power consumption model for the CE mechanism in LEO satellite-based IoT networks. The basic power consumption model for different modes of the NB-IoT terminal follows 3GPP’s technical report TR 45.820 [24]. The CE mechanism during the random access procedure can indeed improve the preamble detection probability. However, it also introduces additional delays and power consumption. Therefore, it is crucial to determine the relationship between preamble repetition of the CE mechanism and power consumption of NB-IoT terminals. As NB-IoT is designed as a low-power and energy-efficient IoT system, the RRC layer plays a crucial role in controlling communication between terminals and satellites over the radio interface to allocate resource blocks. The RRC protocol operates in two states: the RRC Connected state and RRC Idle state. When NB-IoT terminals choose to work, they need to be in the “RRC Connected” state. According to the influence of the CE mechanism, NB-IoT terminals will repeatedly switch between the RRC Idle state and RRC Connected state which causes increased power consumption.

**Figure 5:**
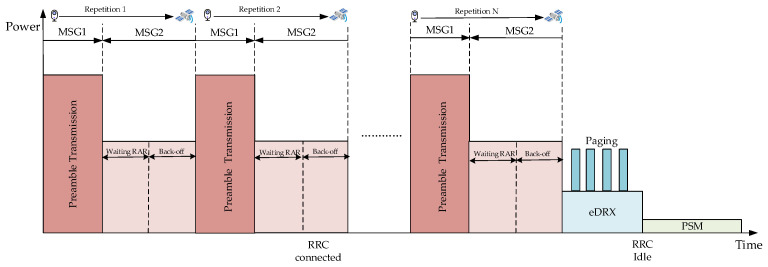
NB-IoT power consumption model.

As shown in Figure 5, we only consider the power consumption arising from the process of repetitions and the back-off state (i.e., RRC Connected state). When the terminal is in the RRC Connected state, we consider two power consumption states. The first is the power consumption of the NB-IoT terminal transmitting the preamble in order to establish a connection and synchronization with the satellite. The second is the power consumption of the terminal receiving the RAR sent by the satellite and waiting for back off for repetition. Namely, we focus on power consumption which is caused by two factors: (1) the repetition of MSG1 due to SINR not reaching the demodulation threshold or preamble collision; (2) the back-off state of MSG2 which is waiting for the RAR window. Thus, combining these two factors with the power consumption model in the 3GPP standard, it can be divided into power consumption during preamble repetition and power consumption in the back-off state and receiving RAR. According to the 3GPP R17 standard file, the power consumption of the back-off state and receiving RAR is the same.

In conclusion, we can derive the power consumption for a successful RA procedure, as shown below:

(24) $$E=N_{\mathrm{rep}}\cdot E_{\mathrm{Trans}}+\left(N_{\mathrm{rep}}-1\right)\cdot E_{\mathrm{connect}}$$

where $E_{Trans}$ is the power consumption during preamble repetition and $E_{connect}$ represents the power consumption in the back-off state and in receiving RAR.

Considering that NB-IoT terminals are usually deployed in hard-to-reach areas, such as mountains and oceans, power consumption requirements are more stringent in these scenarios. Therefore, finding an efficient CE mechanism can strike a balance between power consumption and RACH success probability. This balance ensures the most appropriate relationship of RACH success probability and power consumption for different applications in LEO satellite-based IoT networks. For this reason, we combine these two conditions to establish an optimization problem and solve it in the next section.

## 4. Formulation and Solution of the Optimization Problem

In this section, a multi-objective optimization problem is formulated to optimize RACH success probability and minimize the power consumption of NB-IoT terminals in LEO satellite-based IoT networks. To obtain the appropriate CE configuration parameters for the NB-IoT terminal, we employ NSGA-II to solve this optimization problem. Moreover, we find the optimal parameters in the Pareto-front solution set to meet the RACH success probability requirement. According to different application requirements, we also design a random access parameter configuration method to minimize the power consumption under the constraint of the RACH success probability requirement.

### 4.1. Optimization Problem Formulation

According to the equation of RACH success probability and power consumption, the multi-objective optimization problem can be formulated as follows:

(25) $$\begin{array}{l} (P1):\underset{N_{\mathrm{rep}},W_{\mathrm{Bo}}}{\max}\;\;\;P_{s}\;\\ (P2):\underset{N_{\mathrm{rep}},W_{\mathrm{Bo}}}{\min\;}E\\ s.t.C1:\;T_{\mathrm{msg}1}+RTD+T_{0}+T_{\mathrm{RAR}}+T_{CR}\leq T_{\mathrm{RAO}}<T_{\max}\\ \;\;C2:\;0<N_{\mathrm{rep}}\leq N_{\mathrm{rep}}^{\max}\\ \;\;C3:T_{\mathrm{pre}}\cdot N_{\mathrm{rep}}+W_{\mathrm{BO}}\left(N_{\mathrm{rep}}-1\right)=T_{\mathrm{msg}1}+RTD+\mathrm{StartRAR} \end{array}$$

The objective function P1 denotes the RACH access probability by configuring the number of repetitions and back-off windows. The objective function P2 denotes the power consumption of NB-IoT terminals. The constraint C1 is the time of access that is less than the size of a random access occasion $T_{RAO}$ and the satellite beam coverage time $T_{\max}$ in 3GPP’s technical report TR 38.821. The constraint C2 is the number of repetitions $N_{\mathrm{rep}}$ and must be smaller than the maximum number of repetitions $N_{\mathrm{rep}}^{\max}$. The constraint C3 is the total time of repetitions and back-off should be equal to the sum of the RTD, $T_{\mathrm{msg}1}$, and StartRAR.

This paper has established an optimization model of the NB-IoT CE mechanism and power consumption. It is important to note that $N_{\mathrm{rep}}$ is an integer type in this context. In the next subsection, we will solve this optimization problem and choose the corresponding parameters for different applications.

### 4.2. Solution and Parameter Configurations

To solve this multi-objective optimization problem, we employ NSGA-II to obtain the RACH success probability and power consumption Pareto-front solution set. The goal of our solution method is determining parameter configurations for different applications. Therefore, we need to find the optimal parameters in the Pareto-front solution set that are suitable for different applications. Algorithm 1 shows the proposed solution method to obtain the optimal parameter configuration, which consists of two parts. The first part of this algorithm focuses on finding the Pareto-front solution set for different applications. The second part pays attention to the selection of the optimal parameters within the Pareto-front solution set that minimizes the power consumption of the terminal under the constraint of the RACH success probability requirement.

The RACH success probability and power consumption are employed as the objective function within the NSGA-II. Subsequently, the Pareto-front solution set pertaining to terminal performance in LEO satellite-based IoT networks is obtained through a sequence of fast non-dominated sorting, tournament selection, and genetic evolution of the population until the maximum number of iterations is reached.
**Algorithm 1: Parameter Configuration Method****Input:** Number of repetitions $N_{rep}^{\min}$, $N_{rep}^{\max}$; Back-off Window size $W_{BO}$; Random Access Occasion $T_{RAO}$; Restraint condition INF; Population size POP. **Output:** Pareto optimal solution set $C_{tar}$
 Set NSGA-II iteration counter it=0
 Set maximum number of iterations MAX
 %NSGA-II algorithm to find the Pareto solution set for the service scenario % C=fast-non-dominated-sort(C)
 **for**
i=1 to gen
**do**
 $P_{i}=\mathrm{tournament}-\mathrm{selection}(C)$
 Get $Q_{i}=\mathrm{make}-\mathrm{new}-\mathrm{pop}(P_{i})$
 Get $R_{i}=P_{i}\cup Q_{i}$ $F=\mathrm{fast}-\mathrm{non}-\mathrm{dominated}-\mathrm{sort}(R_{i})$
 Set $C_{i+1}=\emptyset \;\mathrm{and}\;t=1$
 Until $\left|C_{i+1}\right|+\left|F_{i}\right|\leq pop$:   $\mathrm{crowding}-\mathrm{distance}-\mathrm{assignment}(F_{t})$  $C_{i+1}=C_{i+1}\cup F_{t}$  t=t+1
 Set $C=C_{i+1}\cup F_{t}\left[1:(pop-\left|C_{i+1}\right|)\right]$
**end for**
%Find the parameters $C_{tar}$ that match the business scenario% Set $INF(N_{rep}^{\max},T_{\max},W_{BO})$
**for**
i=1 to POP
**do**
 **if**
$C_{tar}\left[N_{rep},W_{BO}\right]\leq MAX$
**then**
  **if**
$C_{tar}\left[N_{rep},W_{BO}\right]\leq MIN$
**then**
   $MIN=C_{tar}\left[N_{rep},W_{BO}\right]$   $C_{tar}=C_{tar}[N_{rep},W_{BO}]$
  **end if**
 **end if**
**end for**

## 5. Simulation and Analysis

In this section, we use the NS-3 simulator and MATLAB to simulate the performance of the proposed ACE method. The satellite parameters and the NB-IoT terminal power consumption values refer to 3GPP’s technical report TR 38.821 [25]. To illustrate the effectiveness of the proposed ACE method, we employ the parameters of CE in 3GPP’s technical report TR 36.763 and the Particle Swarm Optimization (PSO) algorithm for terrestrial-based NB-IoT networks as the benchmark problems. The specific parameters of the simulation are shown in Table 2.

**Figure 6:**
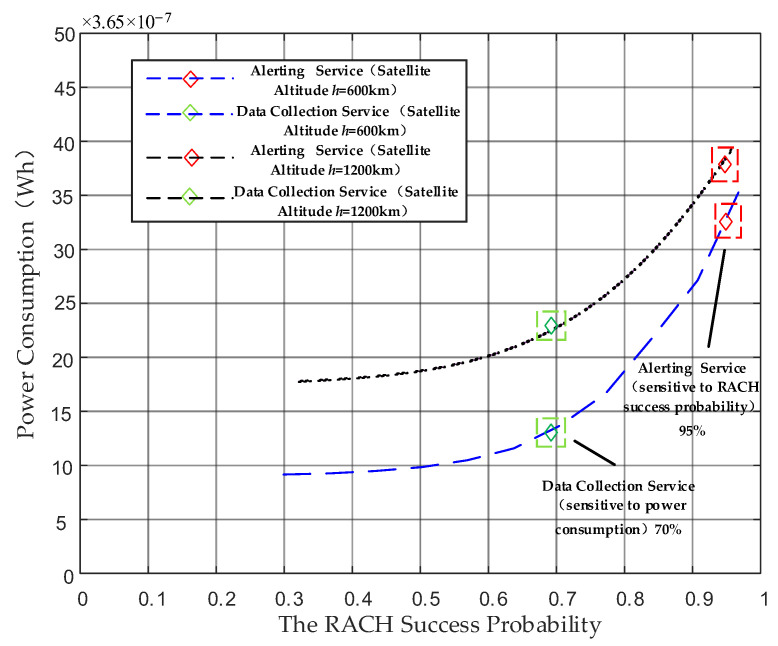
NSGA-II Pareto front for different traffic models.

Figure 6 shows that in the feasible solution set within the Pareto front, the random access parameters of the NB-IoT terminal can be configured based on different application requirements. For instance, lower power consumption is crucial to data collection services with a sensitivity towards power consumption. Conversely, for alerting businesses that prioritize high RACH success probability in real time, substantial repetitions are necessary to ensure successful preamble transmission. For this kind of terminal, the parameter configuration should align with the maximum values present within the feasible solution set. Moreover, the parameter configuration of the proposed ACE method is shown in Table 3. For two different satellite altitudes, this article presents two different feasible solution sets to meet the requirement of access.

For satellites at different altitudes, their parameter configurations can be found in Table 3. This article mainly focuses on the two LEO satellite altitudes recommended by 3GPP and analyzes the trade-off parameter configuration for the corresponding altitudes and service. For satellites at an altitude of 600 km and with alerting services, the requirement for RACH success probability is above 95%. For this type of NB-IoT terminal, power consumption is not the primary indicator and we need to focus on ensuring RACH success probability. Therefore, compared to data collection services at the same satellite altitude, the maximum number of retransmissions must reach 12 to achieve the goal of RACH success probability. And it also has a shorter back-off window size to ensure that the terminal retransmits within the beam coverage time. At a satellite altitude of 1200 km, a long delay will increase the probability of repetition failure. Then, a larger maximum number of repetitions needs to be set at this altitude and the back-off time window needs to be increased to 2048 ms to ensure successful repetition after the back-off state.

### 5.1. Performance of the Proposed ACE Method with Different Demodulation Thresholds

**Figure 7:**
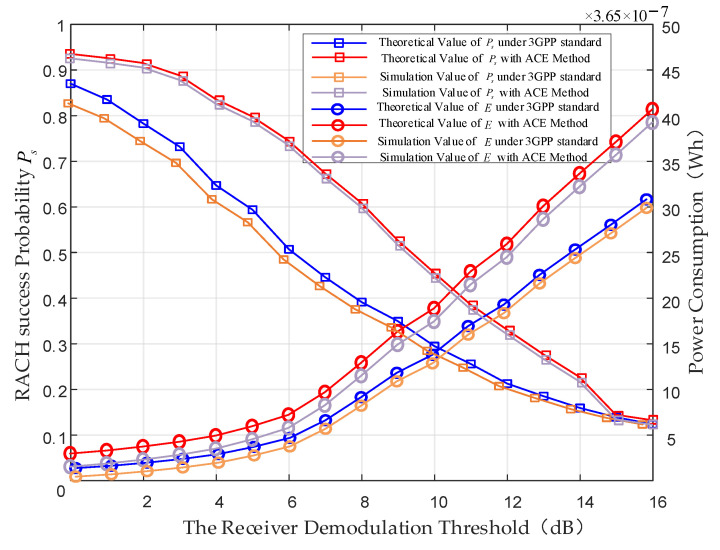
RACH success probability and power consumption performance vary with the demodulation threshold SINRs [24].

**Figure 8:**
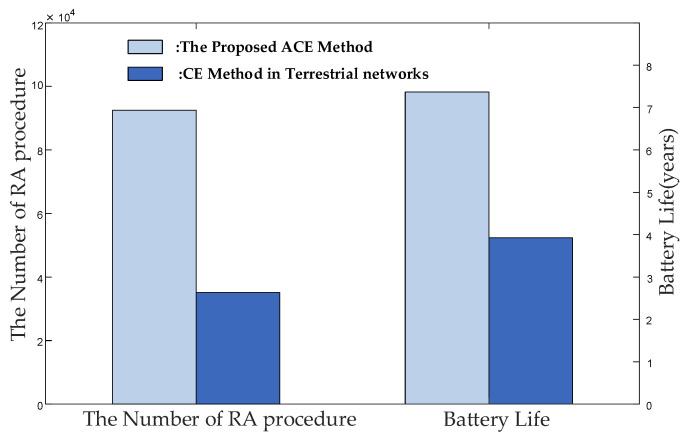
The total number of RA procedures and battery life of NB-IoT terminals with different CE Method.

This section simulates and analyzes the RACH success probability and power consumption for NB-IoT terminals in different SINRs with the parameters in 3GPP Release 17 and optimized parameters. To validate the accuracy of the results obtained through numerical methods, we conducted Monte Carlo simulations to obtain the performance and performed a comparison with a theoretical analysis. Figure 7 indicates a consistent alignment between the simulation curve and the results from the theoretical analysis. Meanwhile, Figure 7 shows the RACH success probability and power consumption comparison of the 3GPP Release 17 parameters and optimized parameters. It is evident that at the same SINR threshold, the RACH success probability with the optimized parameters is higher than that with the original 3GPP parameter configuration. This improvement is attributed to the smaller repetitions and the optimized back-off window size tailored for the long delay of the LEO satellite. The proposed ACE method prevents NB-IoT terminals from having excessive repetitions within a random access occasion and ensures that other terminals can acquire access successfully. This ACE method mitigates the avalanche effect of random access failures, leading to a significant improvement in RACH success probability. Additionally, it reduces the power consumption of NB-IoT terminals. In addition, it is essential to evaluate the performance of the ACE method throughout the entire lifespan of NB-IoT terminals. This article uses the NB-IoT terminal battery standard published by 3GPP for calculations. As shown in Figure 8, if we directly use the CE parameters of the terrestrial network in the LEO satellite-based network, it will result in a significant decrease in battery life.

### 5.2. Performance of the Proposed ACE Method with Different Numbers of Terminals

**Figure 9:**
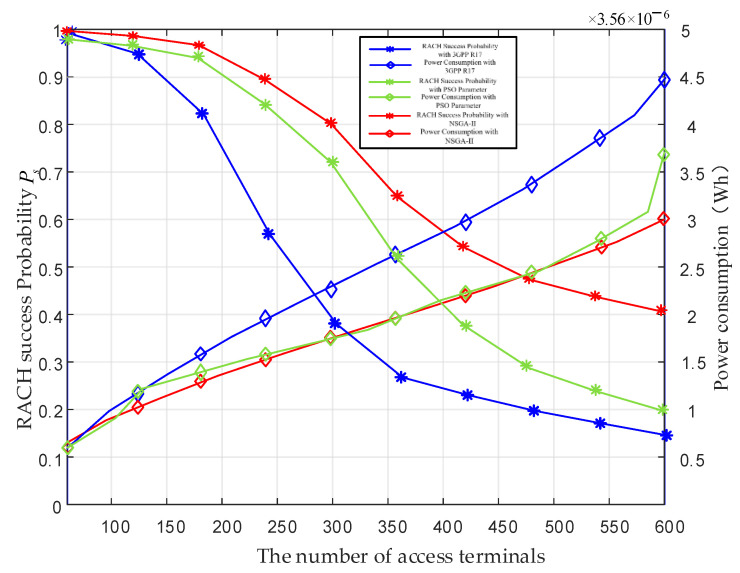
RACH success probability and power consumption performance vary with the NB-IoT terminal load [22,24].

Figure 9 illustrates the RACH success probability and power consumption under different NB-IoT terminal load conditions. According to the specifications in the 3GPP document, the density of NB-IoT terminals in rural areas is set at 0.1 devices/km^2^, with a beam coverage radius of 45 km. It can be calculated that the number of terminals within the beam coverage range is approximately 600–700. Setting the simulation terminal quantity based on this number is reasonable. From this figure, with a growing number of terminals, the collision probability of the preamble increases and leads to a continuous decline in RACH success probability. Meanwhile, the power consumption of NB-IoT terminals also rises. However, the NSGA-II jointly optimized system with two sets of parameters achieves an improvement in RACH success probability. In addition, the proposed ACE method consumes less power and makes it more suitable for LEO satellite-based IoT networks. This is accomplished by reducing the maximum number of repetitions and shortening the size of the back-off window. As can be seen, the proposed ACE method can meet the demands of RACH success probability and power consumption in LEO satellite-based IoT networks.

### 5.3. System Performance with Different Numbers of Terminals

**Figure 10:**
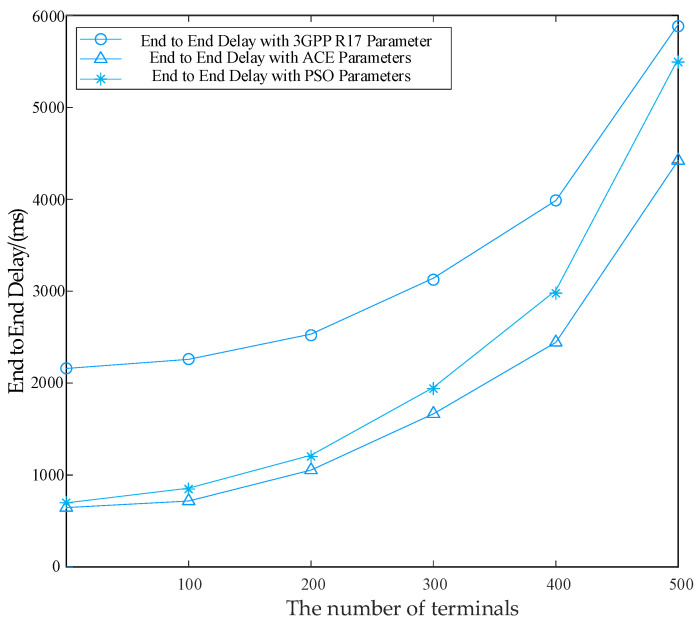
End-to-end delay with different NB-IoT terminal parameter configurations [22,24].

Figure 10 shows the results for the end-to-end delay using the NB-IoT parameter configuration of the 3GPP standard parameters, PSO algorithm optimized parameters, and proposed ACE parameters in the NS-3 simulator. From this figure, with a growing number of terminals, end-to-end latency is on the increase. For configurations with the 3GPP R17 standard, an increasing delay can be noted during the whole communication process. This is because the maximum number of repetitions of the 3GPP standard parameters can reach 128, and excessive repetitions have caused an avalanche effect which results in a significant decrease in RACH success probability. With the proposed ACE parameters, there is a significant decrease in end-to-end delay, which is due to the appropriate size of repetitions and back-off window sizes. Both of these reduce the occurrence of repeated collisions.

**Figure 11:**
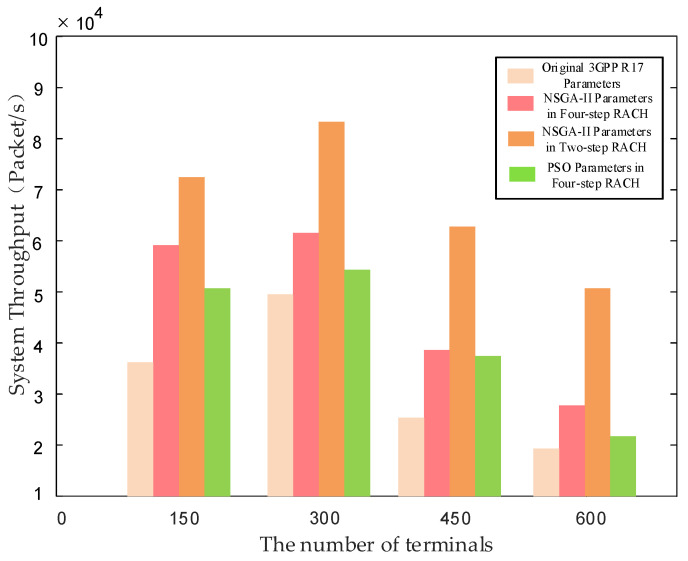
Throughput performance with different NB-IoT terminal parameter configurations [22,24].

Figure 11 shows the throughput comparison of the 3GPP standard parameters, PSO algorithm optimized parameters, and NSGA-II algorithm parameters. In order to make the simulation more realistic, we utilized the NS-3 system-level simulator. By modifying the LTE-LENA module and Channel module, the NB-IoT protocol stack and LEO satellite channel model was implemented. Finally, through date packet simulation, the overall system throughput within the beam coverage time was obtained. It was observed that as the number of terminals increased, the system throughput showed an initial increase followed by a decrease. When the number of NB-IoT terminals is in the range [150, 300], the throughput of the system is on the rise and is always increasing. However, the performance of system throughput gets worse in the range [300, 600]. This trend is attributed to the fact that NB-IoT has only 48 subcarriers. The collision of preamble leads to more repetitions and the avalanche effect. However, the four-step random access NB-IoT terminals with NSGA-II optimized parameters exhibited an improvement of approximately 34–62% in system throughput compared to the NB-IoT terminals with the 3GPP R17 parameters.

Furthermore, combined with the two-step random access proposed in 5G NR, the overall system throughput was further enhanced by reduced delay. However, due to the fact that two-step random access has stricter requirements in the RSRP and requires modifications to a LEO satellite, it is unsuitable to be considered a viable solution now. Therefore, this approach can be considered as a research direction for future LEO satellite-based IoT networks.

## 6. Conclusions

In this article, we analyze the RACH success probability under the CE mechanism in the LEO satellite-based IoT network based on stochastic geometry theory. On the basis of a power consumption model of NB-IoT terminals, an ACE method is proposed to meet the requirement of different applications. Numerical results show that the maximum repetition number of the preamble and back-off window size have a great influence on the system performance, and their value ranges should be set within [4, 18] and [0, 2048] to meet different requirements in LEO satellite IoT networks. The ACE method can effectively enhance the RACH success probability while concurrently reducing power consumption when NB-IoT terminals are engaged in diverse demands. The related conclusions are of practical guidance for the future deployment of NB-IoT terminals in NTN scenarios. However, in the random access procedure, the damage caused by the LEO satellite channel to Msg3 and Msg4 was not considered. The contention resolution results of Msg3 and Msg4 for the random access process are equally important. Furthermore, the resource allocation and scheduling process in the RA procedure need to be studied in detail. Further works on the influence of Msg3 and Msg4 on the RA procedure need to be discussed such as the problem of outdated resource allocation in LEO satellite-based IoT networks.

## Figures and Tables

**Table 1:** The number of repetitions in NB-IoT NPRACH.

Type of Link	Physical Random Access Channel	Number of Repetitions
Uplink	NPRACH	{1, 2, 4, 8, 16, 32, 64, 128}

**Table 2:** Simulation parameters.

Parameter	Value
Transmit power of terminal $p_{s}$ Receive power of terminal	200 mW 90 mW
RRC_Connected power of terminal $E_{connect}$ Battery capacity	90 mW 5 Wh
Satellite orbit altitude h	600 km, 1200 km
Sat Rx max Gain $G_{0}$	24 dBi
Sat beam diameter R	90 km
G/T	−4.9 dB/K
Average power of the line-of-sight path $\Omega_{s}$	0.825
Fading severity parameter $m_{s}$	10
Average power of the scattered path 2b	0.252
Noise density $\sigma^{2}$	−174 dBm/Hz
Density of active IoT devices λ	0.1 device/${\mathrm{km}}^{2}$
Number of sub-carriers	48
Maximum Number of repetitions $N_{\mathrm{rep}}^{\max}$ Back-off Window size $W_{BO}$ Time of RAO $T_{RAO}$ Packet Size Simulation time	{4, 12}, {8, 18} [0, 256·$2^{j}$], j ∈ {0, 3} ms 640 ms 125 byte 15 min

**Table 3:** The proposed Adaptive Coverage Enhancement method.

Type of Service	Maximum Number of Repetitions	Back-Off Window Size
Alerting Service (Satellite Altitude 600 km)	12	[0, 512] MS
Data Collection Service (Satellite Altitude 600 km)	4	[0, 1024] MS
Alerting Service (Satellite Altitude 1200 km)	18	[0, 2048] MS
Data Collection Service (Satellite Altitude 1200 km)	8	[0, 1024] MS

## Data Availability

Data are contained within the article.

## Abbreviations

NB-IoT	Narrowband Internet of things
LEO	Low Earth Orbit
CE	Coverage Enhancement
RACH	Random Access Channel
NGSA-II	Non-dominated Sorting Genetic Algorithms-II
ISL	Inter-Satellite Link
D2D	Direct to Device
NTN	Non-terrestrial Network
MAC	Medium Access Control
RLC	Radio Link Control
PDCP	Packet Data Convergence Protocol
RRC	Radio Resource Control
DMRS	Demodulation Reference Signal
GNSSs	Global Navigation Satellite Systems
HARQ	Hybrid Automatic Repeat Request
SINR	Signal-to-Interference-plus-Noise Ratio
TA	Time Advance
RSRP	Reference Singal Receiving Power
PDF	Probability Distribution Function
LOS	Line of Sight
NLOS	Non-line of Sight
RAR	Random Access Response
RTD	Round Trip Delay
PSO	Particle Swarm Optimization
3GPP	3rd Generation Partnership Project
